# Delayed Diagnosis of Pulmonary‐Renal Syndrome Presenting as Chronic Hemoptysis Leading to ESRD

**DOI:** 10.1155/crpe/2910400

**Published:** 2026-07-06

**Authors:** Kalie Nuss, Katiana Garagozlo, Lilliana Barillas-Arias

**Affiliations:** ^1^ Department of Pediatrics, University of South Florida Morsani College of Medicine, Tampa, Florida, USA, usf.edu; ^2^ Department of Pediatrics, Division of Pediatric Pulmonology, University of South Florida Morsani College of Medicine, Tampa, Florida, USA, usf.edu; ^3^ Department of Pediatrics, Division of Pediatric Rheumatology, Johns Hopkins All Children’s Hospital, Saint Petersburg, Florida, USA, hopkinsallchildrens.org

## Abstract

This case demonstrates the importance of recognizing the various presentations of MPA in pediatric patients and highlights the need for more documentation and discussion of MPA in the current literature in order to increase awareness of MPA and provide possible basis for future diagnostic criteria. MPA is known to primarily affect the kidneys and present with a variety of symptoms. Though pulmonary involvement is reported in some cases of MPA, it typically does not produce overt symptoms. In our case, chronic hemoptysis was the primary presentation of our patient’s MPA, which introduces a unique consideration for clinical diagnosis. This atypical presentation led to a delay in diagnosis, which allowed our patient’s disease to progress to ESRD. As more cases of MPA arise, it is essential to consider their presentations in order to assess and possibly formulate future diagnostic criteria which may help allow physicians to diagnose this condition earlier and avoid life‐long complications in the pediatric population.

## 1. Introduction

A 15‐year‐old female with no significant past medical history presents to a Pediatric Pulmonology clinic for chronic hemoptysis. Mother provided verbal consent for inclusion in this case report. Four months prior to diagnosis, the patient had experienced a respiratory illness which was thought to be the flu which was managed by her pediatrician. During the course of the three to 4 months prior to diagnosis, she had multiple emergency department visits prior to her pulmonary consultation where she was diagnosed with pneumonia via chest radiography and treated with multiple courses of antibiotics (amoxicillin) and bronchodilators which minimally improved her symptoms. During her pulmonary consultation, she endorsed experiencing a teaspoon of bright red blood‐streaked sputum a few times a week for 3 months, associated with a dry cough. The patient endorsed dyspnea with minimal exertion such as walking up one flight of stairs or walking around her bedroom. She also endorsed dizziness, decreased appetite, and significant weight loss of 7 pounds in 1 week. Upon review of pediatrician lab work, she has microscopic hematuria and proteinuria as well as anemia with a hemoglobin of 7.9 g/dL. On examination, she was hypertensive with a blood pressure of 140/84 mmHg, her oxygen saturation was 100%, and her lung exam was unremarkable. Her in‐office spirometry was suggestive of a restrictive pattern. Due to concerns for underlying vasculitic syndrome, she was expeditiously referred to Pediatric Rheumatology and subsequently admitted to the hospital for further workup.

During her initial hospitalization, pertinent lab work included severe microcytic anemia with a hemoglobin of 6.9 g/dL, thrombocytosis, elevated BUN of 27 mg/dL, elevated creatinine of 2.02 mg/dL, hypoalbuminemia, elevated ESRD of greater than 145 with normal CRP, normal C3/C4 and immunoglobulins, elevated urine protein of 710 mg/dL, and an elevated urine protein/creatinine ratio of 5.9 g/day. She had positive antinuclear antibody (ANA) of 1:1280, positive perinuclear antineutrophil cytoplasmic antibody (P‐ANCA) of 1:160, high myeloperoxidase (MPO) antibodies (> 100). Her proteinase‐3 antineutrophil cytoplasmic antibody (PR3‐ANCA), double stranded DNA (dsDNA), and antiglomerular basement antibody were negative. This antibody pattern was most consistent with microscopic polyangiitis (MPA) with renal and pulmonary involvement. Computed tomography (CT) of the chest demonstrated scattered ground glass opacities and pulmonary nodules (Figure 1a,b), which raised concern for an underlying systemic process. She underwent a bronchoscopy which revealed diffuse bloody secretions which was consistent with pulmonary hemorrhage. Infectious workup for pulmonary hemorrhage included negative bronchoalveolar lavage cultures, an indeterminate quantiferon but a negative PPD which ruled out pulmonary tuberculosis.

**FIGURE 1:**
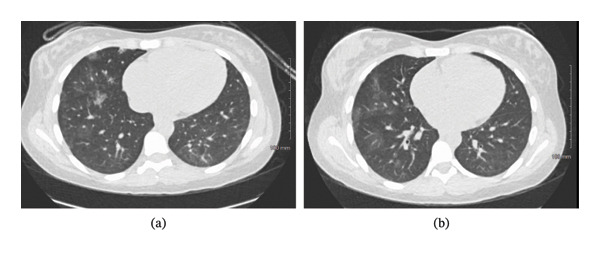
(a, b) Computed tomography of the chest demonstrates scattered ground glass opacities and pulmonary nodules.

Additionally, she underwent a renal biopsy which showed marked diffuse global active necrotizing, sclerosing, and focally crescentic pauci‐immune glomerulonephritis (GN), marked tubointerstitial inflammation and interstitial fibrosis consistent with rapidly progressive GN. Due to significant systemic inflammation in multiple organ systems with infectious etiology less likely, the patient was treated (induction phase) with pulse dose intravenous steroids (1000 mg/kg/day for 5 days). Due to the severity of organ involvement (rapidly progressive GN and pulmonary capillaritis), a multidisciplinary team consisting of rheumatology, nephrology, and pulmonology recommended intravenous cyclophosphamide for induction at 500 mg/m^2^/dose multiplied by 1.6 m^2^ dosing by body surface area (BSA) for a total dose of 800 mg. She was continued on oral corticosteroids (60 mg daily) for maintenance therapy. She received a second dose of cyclophosphamide 1 month later. Due to a suboptimal response to cyclophosphamide and systemic steroids alone, rituximab was added to her maintenance regimen 1 month later for dual immunosuppression with anti‐B‐cell therapy. Plasmapheresis was not indicated after discussion with the multidisciplinary team.

Her renal status continued to worsen with a decline in her creatinine to 5.22 mg/dL 1 month after diagnosis. Given her aggressive renal disease, repeat kidney biopsy was performed which showed extensive fibrosis and sclerosis suggestive of end‐stage kidney disease, and the patient initiated on dialysis and was referred to kidney transplant services. Her renal transplant occurred about a year and a half after initial diagnosis.

MPA is a rare type of autoimmune vasculitis that leads to widespread inflammation of vasculature that supplies various organs, most commonly the kidneys. It is classified by the Chapel Hill Consensus Conference nomenclature of vasculitides as a small vessel, ANCA‐associated vasculitis. Per the Chapel Hill Consensus definition, necrotizing GN and pulmonary capillaritis are commonly seen with this form of vasculitis [1]. The most common symptoms of MPA are constitutional, followed by those of renal, gastrointestinal, musculoskeletal, and integumentary origin [2]. Additionally, pulmonary involvement may be present in up to half of the pediatric MPA cases, though it most commonly presents as an incidental finding without symptoms [3]. MPA is highly associated with the presence of serum ANCA and renal histology displaying GN, which are seen in nearly all pediatric cases of MPA [4].

MPA requires a clinical diagnosis that considers patient history, laboratory findings, histology, and imaging, as there are no current established diagnostic criteria for MPA in the pediatric population. In 2005, the Pediatric Rheumatology European Society (PRES) sought to standardize clinical criteria for the diagnosis of several vasculitis diseases in children. With the support of the European League Against Rheumatism (EULAR) and Pediatric Rheumatology International Trials Organization (PRINTO), a large three‐step retrospective and prospective study was undertaken to define diagnostic criteria for several common pediatric vasculitides. In 2008, the EULAR/PRINTO/PRES criteria were finalized to include the diagnoses of Henoch–Schönlein purpura, polyarteritis nodosa, Wegener granulomatosis (now known as granulomatosis with polyangiitis), and Takayasu arteritis [5]. While sharing some common pathologic findings with the other vasculitides described in these criteria, MPA is a separate condition that currently lacks specific diagnostic criteria, which makes it challenging for physicians to correctly identify MPA in its early course. This delay in diagnosis and corresponding delay in treatment contributes to poorer outcomes and increases the risk of complications from MPA in pediatric patients.

In addition to the lack of diagnostic criteria for MPA, there is also no agreed upon management strategy. The current available treatments for MPA in pediatric patients are mainly based on adult clinical trial data. Mainstays of treatment include corticosteroids plus immunosuppressants such as cyclophosphamide or rituximab [6]. However, it was noted that these treatments do not seem as successful in preventing relapse in pediatric patients as they do in their adult counterparts. The treatment for induction includes rituximab or cyclophosphamide plus 1‐2 mg/kg/day of oral prednisone, which varies from the adult patient’s therapy. Rituximab is being used increasingly in the pediatric population due to cyclophosphamide’s side effect profile, and it is typically administered in 4 weekly doses of 375 mg/m^2^. In limited or nonsevere cases of MPA, methotrexate or mycophenolate mofetil may be used instead of rituximab or cyclophosphamide. Maintenance therapy then includes a combination of glucocorticoids and rituximab, azathioprine, mycophenolate mofetil, or methotrexate for 2 years following induction. For relapsing and persistent disease, intravenous immunoglobulin may be beneficial as well, and for cases with severe alveolar hemorrhage or proliferative GN, plasmapheresis should be considered as an adjunct [6].

In our case, a 15‐year‐old female presented with chronic hemoptysis that was initially misdiagnosed as pneumonia. After undergoing several rounds of antibiotic treatment for pneumonia, her symptoms persisted, warranting a thorough investigation of her case. When considering hemoptysis in the pediatric population, there are many etiologies to consider including autoimmune conditions such as Goodpasture syndrome and systemic lupus erythematosus with pulmonary hemorrhage, pulmonary arteriovenous malformations, and associated syndromes such as tuberous sclerosis complex, bronchiectasis, pulmonary tuberculosis, and the pulmonary vasculitides, namely, granulomatosis with polyangiitis and MPA. This broad differential necessitated the need for additional testing. This patient’s pulmonary exam was unremarkable, but her chest CT was concerning for an underlying systemic process, as was her renal biopsy demonstrating GN. Upon further analysis, it was revealed that she had a high p‐ANCA, which helped support the diagnosis of an ANCA‐associated vasculitis such as MPA. Both MPA and GPA as well as Churg–Strauss (CS) are associated with ANCA positivity, but our patient lacked the characteristic upper airway findings and granulomatous inflammation associated with GPA as well as the characteristic eosinophilia associated with CS. Additionally, her renal biopsy was most consistent with the necrotizing GN seen with MPA [6].

While the patient’s laboratory work, imaging, and histology are consistent with the current literature on MPA, it is important to note her unique presentation and age when considering the relevance of this case to the broader field of research. There are currently no pediatric‐specific diagnostic criteria for MPA due to its rarity in this population, and as such, it is important to report new or unique presentations. Of the ANCA‐associated vasculitides, including granulomatous polyangiitis and Churg–Strauss, MPA is significantly less likely to present with pulmonary involvement in the pediatric population [2, 4]. Because of this, MPA can be easily overlooked when considering the differential for chronic hemoptysis, as it was initially in our case. This resulted in a more severe manifestation at the time of diagnosis, which was less responsive to available treatments. Of the sparse literature that exists on MPA in pediatric patients, cases tend to respond well to current treatment options, and very few require dialysis or kidney transplant [7, 8]. However, in our case, due to the severity of the disease upon diagnosis and the rapidly progressive nature of the condition, our patient did not respond sufficiently to corticosteroids, cyclophosphamide, or rituximab, and due to the extensive damage to her kidneys, she required a renal transplant.

## 2. Conclusion

This case demonstrates the importance of recognizing the various presentations of MPA in pediatric patients and highlights the need for more documentation and discussion of MPA in the current literature in order to increase awareness of MPA and provide possible basis for future diagnostic criteria. MPA is known to primarily affect the kidneys and present with a variety of symptoms. Though pulmonary involvement is reported in some cases of MPA, it typically does not produce overt symptoms. In our case, chronic hemoptysis was the primary presentation of our patient’s MPA, which introduces a unique consideration for clinical diagnosis. This atypical presentation led to a delay in diagnosis, which allowed our patient’s disease to progress to ESRD. As more cases of MPA arise, it is essential to consider their presentations in order to assess and possibly formulate future diagnostic criteria which may help allow physicians to diagnose this condition earlier and avoid life‐long complications in the pediatric population.

## Funding

The authors have nothing to report.

## Ethics Statement

Our institutional IRB has a Human Subjects Determination survey which we completed. Based on this, it was determined that our proposed activity did not meet the University of South Florida (USF) definition of human subject research and did not need review and approval by the USF IRB.

## Consent

The patient’s mother provided verbal consent for inclusion in this case submission.

## Conflicts of Interest

The authors declare no conflicts of interest.

## Data Availability

The data that support the findings of this study are available on request from the corresponding author. The data are not publicly available due to privacy or ethical restrictions.
